# Association Between Surgical Timing and Perioperative Outcomes in Lumbar Spine Surgery: A Retrospective Cohort Study

**DOI:** 10.3390/jcm15114193

**Published:** 2026-05-28

**Authors:** Mehmet Sahap, Yusuf Furkan Gunes, Muhammed Talha Dasgın, Onder Aydemir, Yasar Unsal

**Affiliations:** 1 Department of Anesthesiology and Reanimation, Faculty of Medicine, Ankara Yıldırım Beyazıt University, Ankara Bilkent City Hospital, Ankara 06800, Turkey; 2 Department of Anesthesiology and Reanimation, Kahta State Hospital, Adıyaman 02600, Turkey; 3 Departmant of Anesthesiology and Reanimation, Ankara Bilkent City Hospital, Ankara 06800, Turkey; m.talhadasgin@gmail.com; 4 Provincial Health Directorate, Konya 42285, Turkey; 5 Department of Neurosurgery, Ankara Bilkent City Hospital, Ankara 06800, Turkey

**Keywords:** spine surgery, surgical timing, intensive care unit, perioperative outcomes, inflammation

## Abstract

**Background/Objectives**: The impact of surgical timing on perioperative outcomes remains a subject of ongoing debate. Although after-hours surgery has been associated with adverse outcomes in various surgical populations, its role in lumbar spine surgery remains unclear. This study aimed to evaluate factors associated with postoperative intensive care unit (ICU) admission in patients undergoing lumbar spine surgery. **Methods**: In this retrospective observational study, 460 patients who underwent lumbar spine surgery were analyzed. Patients were categorized according to the timing of surgery as in-hours or after-hours. Demographic, clinical, intraoperative, and laboratory parameters were compared between the two groups. Multivariable logistic regression analysis was performed to evaluate factors associated with postoperative ICU admission. **Results**: Of the 460 patients, 380 (82.6%) underwent surgery during in-hours and 80 (17.4%) after-hours. The baseline characteristics, including age, sex, and comorbidities, were comparable between the two groups. ICU admission was significantly more frequent in the after-hours group (45.0% vs. 19.5%, *p* < 0.001), whereas the length of hospital stay and intraoperative variables were similar between the two groups. Preoperative laboratory analysis revealed higher inflammatory markers (NLR and SII) and lower albumin levels in the after-hours group (all *p* < 0.05), whereas postoperative values were comparable. In the multivariable analysis, after-hours surgery remained independently associated with ICU admission (adjusted OR 4.82, 95% CI 2.15–10.78, *p* < 0.001). Lower preoperative albumin and higher preoperative NLR demonstrated borderline significance. **Conclusions**: After-hours lumbar spine surgery was associated with a significantly increased rate of ICU admission, even after adjustment for patient characteristics, surgical urgency, and clinical indications. These findings suggest that surgical timing may contribute to increased perioperative resource utilization and highlight the potential role of system-level and organizational factors in influencing perioperative outcomes.

## 1. Introduction

Lumbar spine surgery is among the most commonly performed procedures for treating a wide spectrum of pathologies, ranging from degenerative diseases to traumatic injuries and infections. With the increasing surgical volume in recent years, there has been a growing interest in identifying factors associated with perioperative outcomes in this patient population [1]. Complications in these patients are closely associated with prolonged hospital stays, increased healthcare costs, and mortality [2]. Therefore, the accurate identification of perioperative risk factors is critical for improving patient safety and optimizing clinical outcomes.

The impact of surgical timing—defined as the time of day at which the operation is initiated or whether it is performed during or after-hours—on perioperative outcomes has emerged as an important area of investigation. Studies conducted across various surgical disciplines have suggested that procedures performed in the afternoon or nighttime hours may be associated with increased rates of complications, higher intensive care utilization, and increased mortality [3,4,5]. However, the underlying mechanisms of this association remain unclear, and the available evidence is inconsistent.

In the context of spinal surgery, several studies have demonstrated that after-hours emergency procedures may be associated with adverse perioperative events [6,7]. Nevertheless, it remains uncertain whether this association reflects a direct effect of surgical timing or is attributable to the fact that patients undergoing surgery during these periods often present with more severe clinical conditions. Patients requiring urgent surgical intervention are typically characterized by more advanced neurological deficits, higher comorbidity burden, and greater surgical complexity [8]. This raises the possibility that the relationship between surgical timing and outcomes may, at least in part, be explained by the confounding factors, particularly surgical urgency.

In contrast, procedure-related factors, such as operative duration, are well-established contributors to postoperative complications [9]. Prolonged operative time has been associated with increased blood loss, higher infection risk, and systemic complications and is often considered a surrogate marker of surgical complexity [10,11]. Similarly, the patient’s perioperative physiological reserve, inflammatory response, and hemodynamic stability are important contributors to surgical outcomes [12].

In this context, surgical timing should not be evaluated as an isolated risk factor but rather within a comprehensive framework that includes patient characteristics, surgical complexity, and system-level factors that affect outcomes. However, studies specifically addressing lumbar spine surgery and jointly evaluating surgical timing, patient-related variables, and perioperative variables are limited.

This study aimed to evaluate the impact of surgical timing (in-hours vs. after-hours) on perioperative outcomes in patients undergoing lumbar spine surgery. Furthermore, we aimed to provide a more comprehensive understanding of this relationship by analyzing it in conjunction with surgical urgency (elective vs. emergency) and other patient-related and surgical factors.

## 2. Materials and Methods

### 2.1. Study Design and Ethical Approval

This retrospective observational study was conducted in the Neurosurgery and Anesthesiology and Reanimation Departments of the Ankara Bilkent City Hospital. The study protocol was developed in accordance with the principles of the Declaration of Helsinki and was approved by the Ankara Bilkent City Hospital Clinical Research Ethics Committee (Date: 4 December 2024; Approval No: TABED 1-24-784).

### 2.2. Study Population

**Figure 1:**
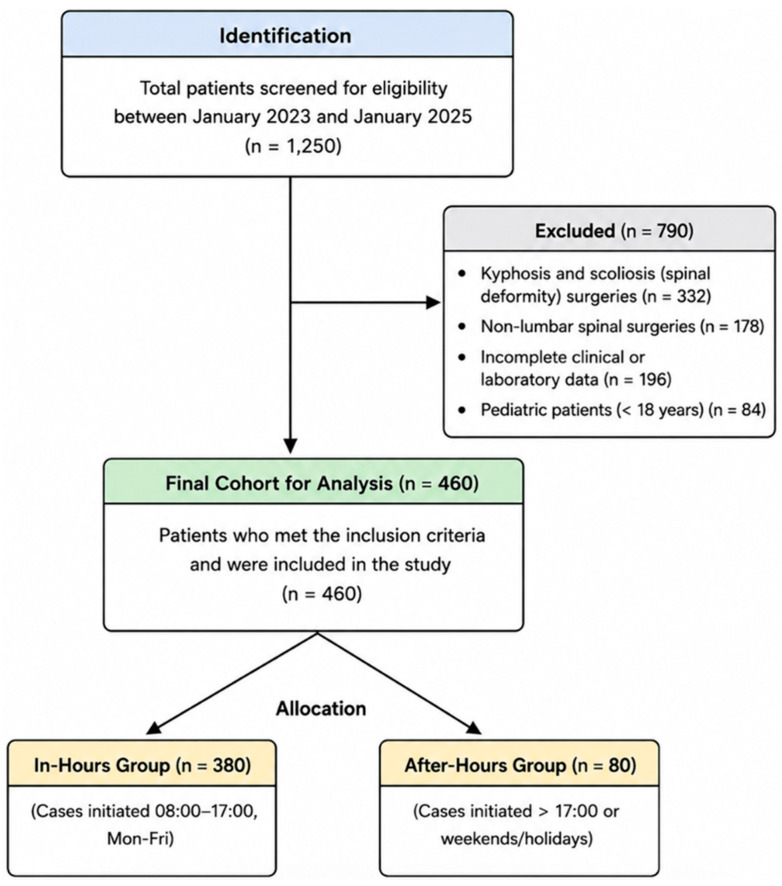
STROBE flow diagram illustrating patient selection and exclusion process.

Adult patients who underwent lumbar spine surgery between January 2023 and January 2025 at Ankara Bilkent City Hospital were retrospectively evaluated. During this period, a total of 1250 patients were screened for eligibility. Following the initial screening, 790 patients were excluded from the study for the following reasons: kyphosis and scoliosis (spinal deformity) surgeries (n = 332), spinal surgeries performed outside the lumbar region (n = 178), missing or incomplete clinical/laboratory data (n = 196), and pediatric patients under 18 years of age (n = 84). Consequently, a final cohort of 460 patients was included in the analysis (Figure 1).

The inclusion criteria were age ≥ 18 years and having undergone lumbar spine surgery under general anesthesia. Patients were categorized into two groups based on the surgical timing: in-hours and after-hours. In-hours surgeries were defined as procedures initiated between 08:00 and 17:00 on weekdays (Monday–Friday). After-hours surgeries included procedures initiated after 17:00 or before 08:00 on weekdays, and all surgeries performed on weekends (Saturday and Sunday) and public holidays. Surgical timing was strictly determined based on the anesthesia start time (the documented time at which anesthesia induction began).

### 2.3. Data Collection

Demographic, clinical, anesthetic, and perioperative data were retrospectively obtained from the hospital electronic medical record system. The demographic variables included age and sex of the patients. Clinical assessment included the American Society of Anesthesiologists (ASA) physical status classification, comorbidities (hypertension, diabetes mellitus, coronary artery disease, chronic pulmonary disease, and other coexisting conditions), and smoking status. The surgical and anesthetic variables included surgical urgency, type of surgery, type of general anesthesia, duration of anesthesia, and patient’s positioning. The intraoperative variables included the use of mannitol, steroids, and opioids; total fluid administration; urine output; estimated blood loss; fluid balance; presence and amount of blood transfusion; use of vasopressors/inotropes; intraoperative hypotension; and intraoperative complications. Laboratory parameters were analyzed in both the preoperative and postoperative periods. These included urea, creatinine, glomerular filtration rate, albumin, hemoglobin, neutrophil and lymphocyte counts, red cell distribution width (RDW), platelet count, and mean platelet volume (MPV) levels. In addition, the following inflammatory indices were calculated: neutrophil-to-lymphocyte ratio (NLR), platelet-to-lymphocyte ratio (PLR), systemic immune–inflammation index (SII), and RDW-to-albumin ratio (RAR).

### 2.4. Outcome Measures

The primary outcome of this study was intensive care unit (ICU) admission. The decision for postoperative ICU admission was made by the attending anesthesiologists and neurosurgeons based on a clinical algorithm that included: ASA physical status ≥ III, significant intraoperative hemodynamic instability, estimated blood loss > 1000 mL, or the need for advanced surveillance during the after-hours period when ward staffing is reduced. The secondary outcomes included in-hospital mortality and length of hospital stay (LOS).

### 2.5. Statistical Analysis

Statistical analyses were performed using IBM SPSS Statistics software, version 25.0. Continuous variables are expressed as mean ± standard deviation or median (interquartile range) according to the data distribution, whereas categorical variables are presented as numbers and percentages. Normality was assessed using the Kolmogorov–Smirnov test. For group comparisons, the independent samples *t*-test or Mann–Whitney U test was used for continuous variables, and the chi-square test or Fisher’s exact test was used for categorical variables, as appropriate.

To identify variables independently associated with ICU admission, variables with a *p*-value < 0.1 in the univariable analysis were included in a multivariable logistic regression model. The results are reported as odds ratios (ORs) with 95% confidence intervals (CIs). Model calibration was assessed using the Hosmer–Lemeshow goodness-of-fit test. A two-sided *p*-value of <0.05 was considered statistically significant. To control for the potential confounding effect of case severity and urgency, surgical urgency (elective vs. emergency) and surgical indications were included as covariates in the multivariable logistic regression model.

## 3. Results

During the study period, 1250 patients were screened, of whom 460 met the inclusion criteria and were included in the final analysis (Figure 1). Among them, 380 (82.6%) underwent surgery during working hours and 80 (17.4%) after-hours. There were no significant differences between the groups in terms of age (53.9 ± 15.0 vs. 52.9 ± 15.0 years, *p* = 0.556), sex distribution (*p* = 0.555), ASA physical status categorized as low-risk (I–II) versus high-risk (III–IV) (*p* = 0.645), or overall comorbidity burden (*p* = 0.311). However, surgical urgency differed significantly between the groups, with all after-hours procedures being emergency operations (*p* < 0.001). Surgical indications also differed significantly, as patients undergoing after-hours surgery more frequently presented with cauda equina syndrome, traumatic instability, and infection-/tumor-related conditions, whereas degenerative disease predominated in the in-hours group (*p* < 0.001). The duration of anesthesia was significantly longer in the in-hours group compared with the after-hours group (275.4 ± 108.2 vs. 247.6 ± 84.3 min, *p* = 0.031). The baseline characteristics of the study population are summarized in Table 1.

ICU admission was significantly more frequent in the after-hours group, with more than a twofold increase compared to the in-hours group (45.0% vs. 19.5%; *p* < 0.001). There was no significant difference in the length of hospital stay between the groups (*p* = 0.261). Intraoperative hypotension showed a trend toward a higher incidence in the in-hours group, although this did not reach statistical significance (31.6% vs. 20.0%, *p* = 0.054). No significant intergroup differences were observed in vasopressor/inotrope use, intraoperative blood loss, blood transfusion, or intraoperative complications (all *p* > 0.05). The detailed perioperative outcomes are shown in Table 2.

Preoperative laboratory analysis revealed that patients in the after-hours group had significantly lower albumin levels and higher inflammatory markers than those in the in-hours group. Specifically, preoperative albumin was lower (44.00 [40.00–46.00] vs. 45.00 [43.00–47.00], *p* = 0.003), while both NLR (2.82 [1.90–4.37] vs. 2.17 [1.65–3.09], *p* < 0.001) and SII (782.72 [478.83–1191.65] vs. 567.99 [383.43–887.64], *p* < 0.001) were significantly higher. Preoperative hemoglobin and creatinine levels were similar between the groups (both *p* > 0.05). In contrast, postoperative laboratory parameters, including hemoglobin, albumin, creatinine, NLR, and SII, were comparable between the two groups (all *p* > 0.05). All laboratory findings are summarized in Table 3.

Due to the low frequency of mortality events, which precluded a reliable multivariable model for death, multivariable logistic regression analysis was focused on ICU admission as the primary outcome. After adjustment for potential confounders, including surgical urgency and clinical indications, after-hours surgery remained associated with ICU admission (aOR 4.82, 95% CI 2.15–10.78, *p* < 0.001). Higher ASA category (III–IV), longer anesthesia duration, and intraoperative hypotension were likewise associated with increased ICU admission (all *p* < 0.05). Lower preoperative albumin levels and higher preoperative NLR demonstrated borderline associations with ICU admission (*p* = 0.084 and *p* = 0.051, respectively). Increasing age was inversely associated with ICU admission (aOR 0.97, *p* = 0.008). Model calibration was acceptable (Hosmer–Lemeshow *p* = 0.641). The results of the multivariable analysis are summarized in Table 4.

## 4. Discussion

In this study, we evaluated the impact of surgical timing on perioperative outcomes in patients who underwent lumbar spine surgery. The main findings were as follows: ICU admission was significantly higher in patients who underwent surgery after-hours; this association persisted in multivariable analysis, suggesting that surgical timing may be associated with increased ICU utilization; and patients who underwent after-hours surgery exhibited a higher preoperative inflammatory burden and lower physiological reserve.

One of the most striking findings of our study was the significantly increased rate of ICU admission in patients who underwent surgery after-hours. This difference remained significant even after multivariable adjustment, indicating that the observed association was not entirely accounted for by the measured patient-related and intraoperative variables included in our model. The effect of surgical timing on perioperative outcomes has been increasingly investigated in recent years. Several studies across different surgical disciplines have reported that procedures performed after hours or at night are associated with higher complication rates, increased ICU utilization, and higher mortality [13,14,15,16]. These findings are often attributed to system-level factors such as reduced staffing, increased fatigue, and limited access to resources during off-hours [17]. Our findings are consistent with the existing literature and suggest that the association between after-hours surgery and ICU utilization may not be solely attributable to surgical urgency or measurable disease severity markers.

Another important aspect of our study was the comprehensive evaluation of the baseline patient characteristics. No significant differences were observed between the groups in terms of age, sex, or comorbidities. Although higher ASA categories were more frequent in the after-hours group when assessed in detail, this difference disappeared when ASA was categorized as low risk (I–II) or high risk (III–IV). This finding suggests that the observed imbalance may be driven by a smaller subgroup of high-risk patients rather than a generalized increase in disease severity.

Notably, patients who underwent after-hours surgery demonstrated significantly higher preoperative inflammatory marker levels (NLR and SII) and lower albumin levels. These findings indicate a higher inflammatory burden and reduced physiological reserve in this group of patients than in the others. Systemic inflammation and hypoalbuminemia are well-established predictors of adverse outcomes in various surgical populations. Indeed, previous studies have demonstrated that elevated inflammatory indices, such as NLR and SII, as well as hypoalbuminemia and low prognostic nutritional index (PNI), are strongly associated with increased mortality, major complications, sepsis, ICU requirement, and poor long-term survival [18,19,20]. In this context, the elevated inflammatory markers and lower albumin levels observed in after-hours patients support the notion that this group represents a more biologically and clinically vulnerable population than daytime patients. However, these differences alone do not fully explain the increased rates of ICU admission.

Regarding intraoperative variables, no significant differences were observed between the groups in terms of blood loss, transfusion requirements, vasopressor or inotrope use, or intraoperative complications. Although intraoperative hypotension tended to be more frequent in the in-hours group, this difference did not reach statistical significance. Additionally, the longer anesthesia duration observed in the in-hours group may reflect the higher proportion of complex and elective procedures performed during these hours. Previous studies have shown that patient-related factors, such as age, ASA score, surgical complexity, comorbidities, and preoperative risk profile, are independent predictors of intraoperative hypotension and of perioperative complications [21,22,23]. Postoperative care also plays a critical role. In particular, staffing levels, monitoring quality, and resource availability in the post-anesthesia care unit (PACU) may influence the early detection and management of hemodynamic instability [24].

Taken together, these findings suggest that the increased ICU utilization observed after-hours cannot be explained solely by intraoperative instability or surgical complexity but rather reflects the combined effects of patient selection, preoperative risk burden, and system-level factors. The impact of surgical timing on clinical outcomes has also been examined in the context of surgeon fatigue. In a large-scale study including 498,234 procedures, it was demonstrated that performing the procedure the night before was not associated with increased mortality or major complications but only with minimal changes in operative duration [25]. These findings suggest that the effect of surgical timing on outcomes cannot be explained solely by individual performance or fatigue.

Similarly, in our study, although a significant difference in anesthesia duration was observed between the groups, intraoperative complications, blood loss, and transfusion requirements were comparable. Importantly, despite shorter operative times in the after-hours group, ICU admission rates were significantly higher, indicating that operative duration alone is insufficient to explain clinical outcomes. Collectively, these findings support the notion that the observed association may reflect complex multifactorial mechanisms, particularly system-level and organizational factors, rather than surgical complexity alone.

The mechanisms underlying the association between surgical timing and ICU utilization are likely multifactorial. Reduced staffing levels, limited availability of experienced personnel, and restricted access to ancillary services after hours may influence clinical decision-making and lower the threshold for ICU admission [26]. Circadian variations in performance and fatigue may also contribute to the clinical outcomes [27]. It has been shown that decreased cognitive performance during nighttime hours may adversely affect clinical decision-making processes [28]. Therefore, the differences observed in our study may be attributed not only to patient and surgical factors but also to the broader system-level and organizational dynamics of the healthcare system.

These findings have important clinical implications. The increased ICU utilization associated with after-hours surgery suggests that this patient population may benefit from enhanced perioperative monitoring and structured care. Furthermore, identifying patients with elevated inflammatory markers and low preoperative albumin levels may facilitate more accurate risk stratification and allow targeted perioperative management strategies.

Beyond the immediate perioperative period, the quality of recovery and long-term functional outcomes, such as return to work, are paramount in lumbar spine surgery. Recent studies have highlighted that various clinical and socioeconomic factors significantly influence the time to return to work following surgery for lumbar stenosis [29,30]. While our study primarily focused on ICU admission as a marker of acute perioperative resource utilization, it is plausible that the physiological stress associated with after-hours surgery could also impact these longer-term recovery trajectories. However, our retrospective design did not allow for the evaluation of patient-reported outcomes or functional recovery milestones, which remains an area for future investigation.

This study has several limitations. First, its retrospective design introduces the possibility of residual confounding despite multivariable adjustment. Second, the single-center nature of the study may limit the generalizability of our findings to different healthcare settings. Third, system-level factors such as provider fatigue, specific staffing patterns, and the exact level of experience of the after-hours surgical team could not be directly evaluated. An important methodological consideration is that all after-hours procedures in our cohort were emergency operations, resulting in substantial overlap between surgical timing and surgical urgency. Although surgical urgency and clinical indications were included in the multivariable model, some degree of collinearity between these variables may have persisted. Therefore, the observed “after-hours effect” should be interpreted within the context of emergency surgical care delivery rather than as a purely isolated timing-related phenomenon. Fourth, the low incidence of mortality events (2.4%) precluded a robust multivariable analysis for this specific outcome, which may have led to statistical underpowering regarding predictors of death. While ICU admission serves as a robust proxy for perioperative morbidity and resource utilization, future larger-scale or multicenter studies are needed to evaluate the factors directly contributing to mortality. Finally, our study primarily focused on acute perioperative outcomes. We did not evaluate long-term functional recovery or patient-reported outcomes, such as the time to return to work, which are known to be influenced by various clinical and socioeconomic factors in lumbar spine surgery patients.

## 5. Conclusions

In conclusion, our study demonstrates that after-hours lumbar spine surgery was associated with increased postoperative ICU admission even after multivariable adjustment for surgical urgency and clinical indications. These findings may reflect not only the intrinsic complexity of emergency cases but also broader system-level and organizational factors, such as reduced staffing and a lower threshold for ICU admission during nighttime and weekend care delivery. While immediate perioperative outcomes appear to be influenced by these organizational dynamics, the potential impact on long-term functional recovery and vocational outcomes also warrants consideration. These findings underscore the importance of optimizing after-hours resource allocation and staffing patterns to ensure consistent perioperative care quality across all surgical shifts. Future prospective multicenter studies are needed to further clarify the relationship between surgical timing, perioperative morbidity, and long-term patient-reported outcomes.

## Figures and Tables

**Table 1:** Baseline Characteristics of Patients According to Surgical Timing (In-hours vs. After-hours).

Variables	In-Hours (n = 380)	After-Hours (n = 80)	*p*-Value
Age, years, mean (SD)	53.9 ± 15.0	52.9 ± 15.0	0.556
Sex, n (%)			0.555
Female	205 (53.9)	42 (52.5)	
Male	175 (46.1)	38 (47.5)	
ASA physical status, n (%)			
I–II	285 (75.0)	58 (72.5)	
III–IV	95 (25.0)	22 (27.5)	0.645
Comorbidity, n (%)	276 (72.6)	53 (66.2)	0.311
Hypertension, n (%)	119 (31.4)	24 (30.4)	0.965
Diabetes mellitus, n (%)	74 (19.5)	14 (17.7)	0.831
Coronary artery disease, n (%)	47 (12.4)	8 (10.3)	0.729
COPD/Asthma, n (%)	29 (7.7)	6 (7.6)	1.000
Smoking, n (%)	93 (24.6)	23 (29.1)	0.487
Duration of anesthesia, min, mean (SD)	275.4 ± 108.2	247.6 ± 84.3	0.031
Surgical Urgency, n (%)			<0.001
Elective	249 (65.5%)	0 (0.0%)	
Emergency	131 (34.5%)	80 (100.0%)	
Surgical Indication, n (%)			<0.001
Degenerative disease	249 (65.5%)	0 (0.0%)	
Disc Herniation/Neural Compression	115 (30.3%)	46 (57.5%)	
Cauda Equina Syndrome	1 (0.3%)	10 (12.5%)	
Traumatic Instability	7 (1.8%)	9 (11.3%)	
Infection/Tumor/Other	8 (2.2%)	15 (18.8%)	

*p*-values compare the in-hours and after-hours surgery groups. Continuous variables are presented as mean ± standard deviation (SD) and were compared using the independent samples *t*-test. Categorical variables are expressed as numbers (percentages) and were compared using the χ^2^ test or Fisher’s exact test, as appropriate. ASA: American Society of Anesthesiologists; COPD: Chronic Obstructive Pulmonary Disease; SD: Standard Deviation.

**Table 2:** Perioperative Outcomes According to Surgical Timing (In-hours vs After-hours).

Variables	In-Hours (n = 380)	After-Hours (n = 80)	*p*-Value
ICU admission, n (%)	74 (19.5)	36 (45.0)	<0.001
Length of hospital stay, days, median (IQR)	3 (—)	3 (—)	0.261
Intraoperative hypotension, n (%)	120 (31.6)	16 (20.0)	0.054
Vasopressor/inotrope use, n (%)	22 (5.8)	3 (3.8)	0.645
Intraoperative blood loss, mL, median (IQR)	550 (—)	500 (—)	0.503
Blood transfusion, n (%)	58 (15.3)	10 (12.5)	0.646
Intraoperative complications, n (%)	3 (0.8)	0 (0.0)	0.973

*p*-values compare the in-hours and after-hours surgery groups. Continuous variables are presented as medians (interquartile range, IQR) and were compared using the Mann–Whitney U test. Categorical variables are expressed as numbers (percentages) and were compared using the χ^2^ test or Fisher’s exact test, as appropriate. ICU: Intensive Care Unit; IQR: Interquartile Range.

**Table 3:** Preoperative and Postoperative Laboratory Parameters According to Surgical Timing (In-hours vs. After-hours).

Variables	In-Hours (n = 380)	After-Hours (n = 80)	*p*-Value
Preoperative hemoglobin, g/dL	13.90 (12.60–14.90)	13.80 (12.20–14.95)	0.776
Preoperative albumin, g/L	45.00 (43.00–47.00)	44.00 (40.00–46.00)	0.003
Preoperative creatinine, mg/dL	0.78 (0.66–0.89)	0.78 (0.68–0.89)	0.982
Preoperative NLR	2.17 (1.65–3.09)	2.82 (1.90–4.37)	<0.001
Preoperative SII	567.99 (383.43–887.64)	782.72 (478.83–1191.65)	<0.001
Postoperative hemoglobin, g/dL	11.15 (9.70–12.83)	11.40 (9.63–12.90)	0.651
Postoperative albumin, g/L	35.00 (32.00–39.00)	35.00 (31.25–38.00)	0.653
Postoperative creatinine, mg/dL	0.72 (0.59–0.85)	0.73 (0.58–0.87)	0.864
Postoperative NLR	7.44 (4.52–13.10)	8.08 (4.85–12.67)	0.660
Postoperative SII	1677.81 (935.12–3045.05)	1941.71 (1130.89–3251.19)	0.235

*p*-values compare the in-hours and after-hours surgery groups. Continuous variables are presented as medians (interquartile range, IQR) and were compared using the Mann–Whitney U test. NLR, neutrophil-to-lymphocyte ratio; SII, systemic immune–inflammation index; IQR, interquartile range.

**Table 4:** Multivariable logistic regression analysis for ICU admission.

Variables	Adjusted OR (aOR)	95% CI	*p*-Value
After-hours surgery	4.82	2.15–10.78	<0.001
Surgical Urgency (Emergency)	1.95	0.88–4.32	0.098
Age (per year increase)	0.97	0.95–0.99	0.008
ASA III–IV (vs. I–II)	2.34	1.12–4.88	0.024
Duration of anesthesia (per min)	1.01	1.004–1.012	0.006
Preoperative albumin	0.94	0.89–1.01	0.084
Preoperative NLR	1.09	1.00–1.18	0.051
Intraoperative hypotension	1.88	1.02–3.45	0.042

Multivariable logistic regression analysis was performed to evaluate variables independently associated with ICU admission. Variables were selected based on univariable screening and their clinical relevance. The results are presented as adjusted odds ratios (aORs) with 95% confidence intervals (CIs). A two-sided *p*-value < 0.05 was considered statistically significant. Model calibration was assessed using the Hosmer–Lemeshow goodness-of-fit test (χ^2^ = 6.05, *p* = 0.641). ICU: Intensive Care Unit; ASA: American Society of Anesthesiologists; NLR: neutrophil-to-lymphocyte ratio.

## Data Availability

The data are available on reasonable request from the corresponding author due to privacy restrictions.
